# Eco-evolutionary dynamics in a contemporary human population

**DOI:** 10.1038/ncomms15947

**Published:** 2017-07-04

**Authors:** Fanie Pelletier, Gabriel Pigeon, Patrick Bergeron, Francine M. Mayer, Mireille Boisvert, Denis Réale, Emmanuel Milot

**Affiliations:** 1 Département de biologie, Université de Sherbrooke, Sherbrooke, Quebec, Canada J1K 2R1; 2 Département des Sciences Biologiques, Université du Québec à Montréal, Montréal, Quebec, Canada H3C 3P8; 3 Département de chimie, biochimie et physique and Forensics Research Group, Université du Québec à Trois-Rivières, Trois-Rivières, Quebec, Canada G9A 5H7

## Abstract

Recent studies of the joint dynamics of ecological and evolutionary processes show that changes in genotype or phenotype distributions can affect population, community and ecosystem processes. Such eco-evolutionary dynamics are likely to occur in modern humans and may influence population dynamics. Here, we study contributions to population growth from detailed genealogical records of a contemporary human population. We show that evolutionary changes in women’s age at first reproduction can affect population growth: 15.9% of variation in individual contribution to population growth over 108 years is explained by mean age at first reproduction and at least one-third of this variation (6.1%) is attributed to the genetic basis of this trait, which showed an evolutionary response to selection during the period studied. Our study suggests that eco-evolutionary processes have modulated the growth of contemporary human populations.

## Abstract (web summary)

Evolution can occur rapidly enough to influence population growth, though this possibility tends to be discounted for human populations. Here, the authors analyse genealogical records and show that evolution of women’s age at first reproduction contributed significantly to the growth of the île aux Coudres population.

Individual variability in fertility, growth, recruitment, dispersal and mortality affects the population dynamics of any organism^1^. Although population biology has interested scientists for at least two centuries and was key to the development of evolutionary theory^2^,^3^, we still have very little empirical evidence of how evolutionary shifts in inheritable individual differences in traits may affect population growth^4^,^5^. Evolutionary studies are typically interested in changes in mean and variance of quantitative characters or allele distributions, while biodemography studies on animals focus on the effect of life history traits such as survival, reproduction and migration on changes in population size, usually with less emphasis on individual characteristics other than sex, age and developmental stage^6^. Yet, individuals with different genotypes and phenotypes may differ in their capacity to disperse, reproduce and survive^7^, and that variability should affect population growth^8^. Therefore, although ecology and evolution may interact to affect population dynamics^9^,^10^, there is as yet little quantification of the magnitude of those interactions in nature or in human populations.

Recent studies suggest that selection on phenotype and life history is widespread in modern human populations^11^,^12^. There is mounting evidence that modern humans have adapted to differences in local environments, including dietary practices, local climatic conditions, pathogens and pollution^13^. Those studies suggest that interaction between ecology and evolution might exist and could even be widespread in human populations. Here, we quantify the importance of evolutionary changes in age at first reproduction in women on population growth in a contemporary human population where an evolutionary change for this trait was previously documented^11^.

We used the population Register of a contemporary population founded in 1721 on île aux Coudres, Québec, Canada, on the St. Lawrence River, approximately 80 km northeast of Québec city. The population Register was established by linking the civil records of birth, death and marriage contained in parish books (Methods, Supplementary Note 1). This linkage enables the reconstruction of family records. In this study, we focused on women born between 1772 and 1880, for which we had a complete record from birth to death. We filtered couples retained in our analyses as described in the Methods, to limit biases that may arise from incomplete knowledge of the full reproductive history for some couples. Based on censuses, the île aux Coudres population underwent three demographic phases between its foundation and the 1950s (Supplementary Fig. 1): an initial rapid increase in size was followed by slower growth which then led to a second period of rapid increase. A previous study, over the same time period, reported that women first reproductive event (hereafter named age at first reproduction (AFR)—defined as a mother’s age at the birth of the first child) became earlier through time due to changes in both phenotypic and genetic values of age at first in this population^11^. Building upon this result, we used an individual-based approach that links breeding (BV) and phenotypic values of a trait to individual contributions to population growth^14^. This method quantifies retrospectively the proportion of variance in population growth explained by genetic variation in age at first reproduction. We then evaluated the importance of evolution for population processes by calculating population growth and expected population size if no evolution of AFR had occurred over the study. We show that the predicted population growth in the absence of evolution was significantly lower than the observed one. Thus, without evolution in women AFR the predicted population size is 12% smaller over the study period.

## Results

### Linking lifetime contribution to AFR

**Figure 1:**
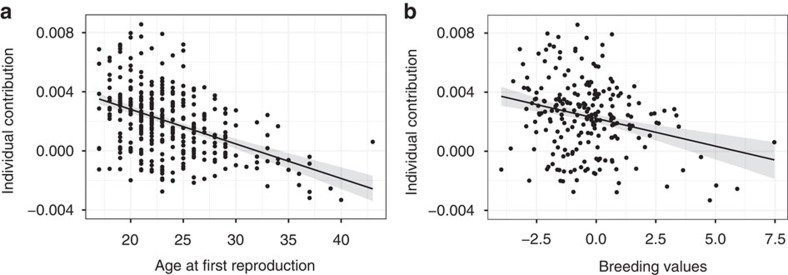
Links between age at first reproduction and lifetime individual contributions. Effect of (**a**) individual age at first reproduction and (**b**) breeding values for age at first reproduction on lifetime individual contributions to population growth (calculated as the sum of yearly individual contributions during a woman’s lifetime, *N*=220), at île aux Coudres for women born from 1772 to 1880. The predictions lines with 95% confidence intervals (generated from a parametric bootstrap) are from linear mixed models presented in Table 1.

Women’s variation in age at first reproduction explained 15.9% of variability in their lifetime individual contribution to population growth over 108 years (Fig. 1a, Table 1). More than one-third of this variation (6.1%) was attributable to variation in breeding values of this trait across generations (Fig. 1b, Table 1). The importance of phenotype or breeding values on lifetime individual contribution did not change with demographic phase, as we found no interaction between trait and demographic phase (Supplementary Table 1). To make sure that our results were robust to the assumptions made when we filtered the data from the Register, we analysed a data set that included women with at least one particularly long birth interval, that is, lying in the upper tail of the distribution (see Methods). The association between breeding values for AFR and lifetime individual contribution was even stronger, explaining 17.3% (versus 6.1%) of variation in population growth (Supplementary Table 2). This larger effect can be explained by the fact that this second data filtering better reflects the full range of expressed fertility among women, thus providing more power to quantify how individual variation contributes to population growth, despite the uncertainty around the true fertility of women with longer birth intervals. The increase in individual contributions with lower BVs for AFR (that is, genetic effects for earlier reproduction) was mainly driven by the contribution of women to population growth through the recruitment of their offspring in the population (Supplementary Table 3). However, women with breeding values for earlier age at first reproduction tended to have a lower lifetime contribution to population growth through survival, suggesting a cost of reproduction (Supplementary Table 3).

### Effect of evolution in AFR on population growth and size

To quantify the impact of evolutionary change in AFR, defined as the change in breeding values over time, on population growth rate, we calculated the growth rate of the population as well as a prediction of what it would have been without evolution. We did this by predicting the individual contribution to population growth as a function of breeding values using the model in Table 1. Predicted growth rate without evolution was obtained by fixing the breeding values of all cohorts to the mean breeding value of the first study cohort, therefore assuming no evolutionary changes (Supplementary Note 2). As this method predicted the lifetime value of individual contribution, we back transformed it into an annual value by dividing it by the number of years each woman was assumed present on the island, distributing it evenly through her lifetime. These new annual predicted contributions to population growth were then used to estimate the difference between the population growth measured from the Register and predicted growth in absence of evolution. A detailed example of the approach is presented in Supplementary Note 2 (with example data in Supplementary Tables 4 and 5 and Supplementary Figs 2 and 3).

**Figure 2:**
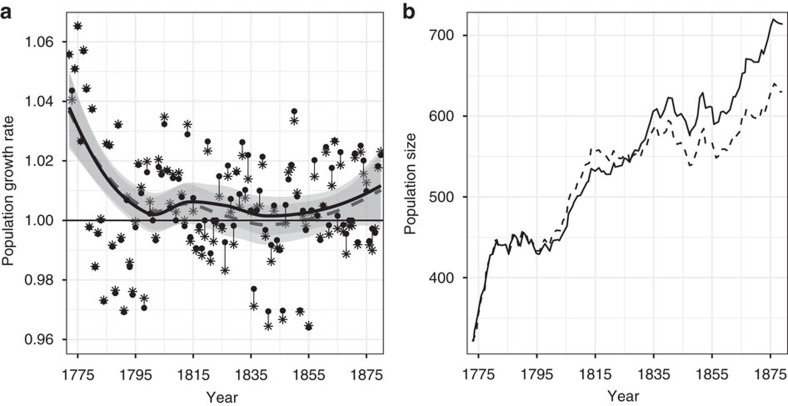
Population dynamics of the île aux Coudres population from 1772 to 1880. In **a** solid circles and full line represent the yearly population growth rates (*N*_*t*+1_/*N*_*t*_) calculated from the Register, while asterisks and the dashed line represent an approximation of the predicted growth rates, in the absence of evolution. The two lines were fitted using loess and are accompanied by their 95% CI (grey areas around the lines). In **b** the full line represents the population size calculated from the Register, whereas dashed line represents population size estimated from the model in absence of evolution.

Predicted population growth in the absence of evolution was lower than the observed one (mean yearly difference of 0.0011 in population growth, paired *t*-test=4.104, d.f.= 108 years, *P*<0.001) with an increasing difference over time (Fig. 2a) due to the temporal decrease in AFR breeding values. This effect corresponds to a difference of approximately 1 child per year for a cohort of 250 women. Thus, without evolution of age at first reproduction, the population would have been about 12% smaller after the 108 years of the study (Fig. 2b). In a context of exponential growth, small evolutionary changes in early cohorts led to a substantial difference in population size on the longer term (Fig. 2b).

## Discussion

As there is accumulating evidence that AFR has evolutionary potential across several modern human populations^15^,^16^, the role of contemporary evolution deserves better attention. Eco-evolutionary dynamics may have non-negligible effects on human population size projections, and thus have important consequences for policies or decisions relying on these projections. The integration of ecology and evolution is therefore fundamental to deepen our understanding of the processes that shape phenotypic and genetic diversity of important traits, such as reproductive traits and susceptibility to disease^4^,^7^. In particular, demographic processes modulate the strength of selection on genetic variants differentially according to the age at which they are expressed^17^,^18^,^19^. Therefore, evolutionary changes in life history traits that affect population growth should also modify selection on genetic variation that correlates with fitness, including variants that may shape health, ageing and lifespan.

Eco-evolutionary dynamics^20^, defined here in the broad sense of an evolutionary change in a trait that causes a change in an ecological variable that then influences selection on the same or some other trait, may also trigger the possibility for feedback loops between demography and evolution in life-history traits, as well as feedback through human population size effects on their domestic and natural environments. Our results suggest that those types of feedback, that is, narrow-sense eco-evolutionary dynamics, are likely to occur in human populations although this remains to be investigated. Here, we assume that if there is cultural transmission, the effect would decay with relationship distance. Thus, we should minimally detect it in closer relatives (for example, mother–daughter) and if there was cultural transmission of AFR we should detect a significant mother effect in our model. Given that we explain negligible variation by this term (see ref. 11) but we do find a large variation explained by the pedigree, it seems unlikely that cultural transmission has an important impact our heritability estimates.

Contemporary evolution can affect ecological processes, including population, community and ecosystem^20^. Thus, understanding the eco-evolutionary dynamics of biological populations—including the human population—depends fundamentally on a mechanistic understanding of how evolutionary forces (for example, natural selection, gene flow) and ecological forces (for example, mortality and disease) interact. A common assumption in human population dynamics, however, is that evolution is very slow and, even when it occurs, it should not have any effects on population processes due to swamping by non-evolutionary factors, including culture, wars, famine, migration and technical advances. Our results challenge this classic view by showing that the tripling in size of the population over 108 years can be partly explained by genetic changes in age at first reproduction that occurred over the same period. Our study is one of very few outside the lab showing that ongoing adaptation can have a measurable effect on population growth. It also illustrates the importance of eco-evolutionary dynamics in human populations.

## Methods

### Description of île aux Coudres database

The population Register links the acts of baptism, marriage and burial registered in Church parish books of île aux Coudres^21^. The population Register also integrates data from an ethnographic census performed in 1967 (ref. 21). The Register covers the period from the establishment of a first family in 1721–1973 and comprises nearly 8,400 individuals and 2,000 unions. Individual contribution to population growth were calculated using all this information. The data are managed by the ANALYPOP software developed in F.M.M.’s laboratory.

### Data retrieval from the île aux Coudres population Register

Population registers link major life events from sources such as Church or civil records, but do not monitor every individual over their lifetime. For example, individuals born on the island who later emigrated were registered at birth, but not at death. Some people may marry and emigrate before producing any offspring, in which case their fertility (completed family size or number of children born) is unknown. Likewise, a couple who emigrated temporarily may have produced some offspring while away. In such cases, there may be no record of these children in the register, resulting in unusually long interbirth intervals. To account for this situation, we assumed that unusually long gaps according to the historical demography criteria^22^ between (i) marriage and first birth, (ii) two successive births, or (iii) the last birth and the end of reproductive life (menopause) reflected emigration, and excluded all couples that showed at least one of these unusual long intervals in their reproductive history. Thus, reproductive life was considered to end with the death of one spouse or when the wife reached 45 years. Our data set therefore excludes couples that may truly be less fecund, leading to conservative results in heritability estimates and therefore in the contribution of genetic changes in AFR to population growth. This data set included 363 marriages with 3,110 births. To ensure that our results were robust to the previously described assumptions, we also examined a data set that did not exclude couples with at least one long birth intervals. Those couples were reintroduced in the data set following two steps: (1) those whose presence was confirmed in the nominative censuses during the long intervals were integrated in the study and (2) modified criteria were adopted through the analyses of these intervals and then applied to the couples not covered by censuses. This data set included 572 marriages with 4,002 offspring births (these two filters were originally developed in a study on infant mortality in the same population (see refs 11, 23) and below for more detailed information on data filtering).

### Description of data filtering

The population Register records major events (births, marriages and deaths) that occurred on the island and therefore does not necessarily give complete individual histories. As such, the population Register does not directly distinguish between migrants, for whom reproductive life occurred outside of the island (totally or partly) and non-fecund couples. Thus, we had to use a subset of the data available in the Register that included only women for which we had information from birth to death. To do so, we applied a filter to the two datasets previously described that included only women born between 1772 and 1880 to ensure that they had the time to complete their family and died by 1973, when the last records were collected. The first data set, called ‘migration’, that excluded couples who had inter-birth or marriage/first birth intervals that were longer than average, therefore included a sample size of 220 women. The second data set, called ‘sub-fecundity’, that kept couples with long birth intervals, assuming a true low fecundity, included a sample size of 338 women. We conducted analyses on both data sets.

### Individual contribution to population growth

To evaluate the proportion of variance in population growth explained by AFR, we first calculated yearly individual contributions to population growth. This quantity is calculated as the difference between population growth rebuilt from the population Register and population growth calculated excluding the contribution of a focal individual. It describes how each individual contributed directly to population growth over an annual time step^14^.

We calculated population growth rate or equivalently, average fitness (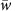), each year as the ratio of the number of individuals in the population at time *t+*1 over the number at time *t* so that 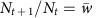 (see ref. 14). We estimated the yearly population size on the island assuming that a person was present between two events (marriage, birth of a child, death) recorded in the Register (see ref. 24 for more details on population size estimation and validation, Supplementary Fig. 1). We then calculated the individual contributions to population growth (*P*_*t*(*i*)_) over an annual time step as the difference between the observed population growth and population growth calculated with the contribution of a focal individual removed. Each individual can contribute to population growth via survival and reproduction; hence we calculated individual contribution as follows: 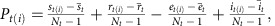
where *s*_*t*(*i*)_, *r*_*t*(*i*),_ *e*_*t*(*i*),_ *i*_*t*(*i*)_ represent the contribution through survival, recruitment, immigration and emigration of individual *i* at time *t*, 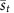, 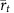, 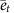 and 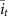 represent mean survival, recruitment, emigration and immigration rates at time *t*, and *N*_*t*_ represents the population size at time *t* (see refs 7, 14). Recruitment was defined as the number of children produced by a woman in year *t* that survived to at least one year of age. Emigration represents individuals that left the island permanently and immigrants were people present in the population register with no birth information. For adults without recorded year of death, we assumed they were on the island until their last record in the population Register or the death of one of their children at 14 years of age or younger. For children with only the year of birth recorded, we assumed they were present on the island until the last familial event recorded on the island (usually birth or death of kin) or until 14 years of age in the case of no familial event. These criteria provide a strong fit of population size estimated from the Register to that obtained from censuses conducted by the Canadian government (Pearson correlation, *r*=0.99, *t* =25.41, *P* <0.001 and ref. 24). As a woman can only have one age at first reproduction, we calculated her lifetime contribution to population growth as the sum of her yearly *P*_*t*(*i*)_. As the immigration and the emigration components of the individual contribution were affected by our assumptions, we did not include them in our calculation of lifetime contribution. Only women with known dates of birth and death were used in the calculation of lifetime individual contributions.

### AFR breeding values

We used the breeding values for AFR (defined as the additive genetic effects on a trait relative to the mean phenotype in the population) estimated in a previous study of the same data sets^11^. These breeding values were estimated in a previous study with a Bayesian quantitative genetic approach that uses information from all the relationships in the genealogy to estimate the expected phenotypic resemblance among individuals that can be ascribed to their genetic relationship (see Methods and refs 11, 25). Briefly, a Bayesian bivariate ‘animal’ model, a kind of mixed effect model using pedigree relationships to infer genetic parameters, is used to estimate the additive genetic variance for AFR and lifetime reproductive success and their genetic correlation, as well as individual breeding values. The model accounted for temporal trends of environmental/cultural origin by entering the year of marriage, inbreeding (quadratic effect), twinning and the familial environment shared by sisters (by entering the parent marriage ID as a random effect). The distribution of AFR was modelled with a Gaussian error structure. See Milot *et al*.^11^ for more details on the animal models and results on quantitative genetic parameters. We then linked both phenotypic and genetic variation in age at first reproduction to population growth as explained below. As a woman can only have one age at first reproduction, we used her lifetime contribution to population growth as the sum of her yearly contributions (that is, lifetime *P*_*t*(*i*)_).

### Statistical analyses

We assessed the proportion of variation in individual contribution (lifetime *P*_*t*(*i*)_) accounted for by the phenotypic and breeding values in age at first reproduction using linear mixed effect models, including women identity as a random effect. In a second set of analyses, we tested whether AFR and BV AFR mostly affect either the recruitment (*R*_*t(i)*_) or the survival (*S*_*t(i)*_) contributions to population growth.

We included birth year as a random effect because as population size increases, the individual contribution will inevitably decrease. Fixed effects included either the age at first reproduction or the breeding values in age at first reproduction. The deviance explained by the variable of interest in these models represents the contribution of the phenotypic or genetic value of age at first reproduction to population growth^7^. To assess whether the importance of age at first reproduction on population growth changed with demographic phases, we also included a variable describing the three demographic phases (see Supplementary Note 1 and Supplementary Fig. 1) and their potential interactions with age at first reproduction or breeding values for age at first reproduction. All analyses were implemented in R version 2.15 (ref. 26). The ‘nlme’ package was used to fit linear mixed effects models.

### Data availability

The data sets generated and/or analysed during the current study are available on request. Researchers interested in accessing the data used in the analyses presented here should contact F.M.M. or E.M.

## Additional information

**How to cite this article:** Pelletier, F. *et al*. Eco-evolutionary dynamics in a contemporary human population. *Nat. Commun.* **8,** 15947 doi: 10.1038/ncomms15947 (2017).

**Publisher’s note:** Springer Nature remains neutral with regard to jurisdictional claims in published maps and institutional affiliations.

## Supplementary Material

Supplementary Information

## Figures and Tables

**Table 1:** Factors affecting lifetime individual contributions to population growth.

	**Variables**	**Coefficients**	**s.e.**	***T*****-values**	***P*** **values**
(a)	Intercept	0.00782	0.00084	9.26	<0.001
	AFR	−0.00023	0.00004	6.57	<0.001
				Deviance explained	15.9%
(b)	Intercept	0.00223	0.00018	12.37	<0.001
	BV for AFR	−0.00038	0.00009	3.99	<0.001
				Deviance explained	6.1%

(a) Effects of age at first reproduction (AFR) and (b) breeding values (BV) for age at first reproduction on lifetime individual contribution to population growth for cohorts of women born in 1772–1880 at île aux Coudres, Canada. Estimates are from linear mixed effects models including year of birth as a random effect. The deviances explained by AFR and BV were estimated by comparing models with and without the variables of interest. The effects of demographic phases and the interactions between phase and AFR or BV were not retained in final models (all *P* >0.108), see Supplementary Table 1 for full models.
