# Supplementary material for: Eco-evolutionary dynamics in a contemporary human population
Source: Nat Commun. 2017 Jul 4;8:15947. doi: 10.1038/ncomms15947 (PMC5500883; doi:10.1038/ncomms15947)
Supplement: Supplementary Information [file ncomms15947-s1.pdf]

Title of file for HTML: Supplementary Information
Description: Supplementary Figures, Supplementary Tables, Supplementary Notes and Supplementary References

Supplementary Table 1. Effects of age at first reproduction and demographic phases on lifetime individual contributions.

	Variables	Coefficients	SE	T-values	P-values
(a)	Intercept	0.00985	0.00260	3.786	<0.001
	AFR	-0.00028	0.00095	2.946	0.004
	Phase 2	-0.00166	0.00277	0.599	0.551
	Phase 3	-0.00567	0.00423	1.338	0.184
	AFR x Phase 2	0.00004	0.00010	0.345	0.731
	AFR x Phase 3	0.00017	0.00017	0.963	0.337
(b)	Intercept	0.00316	0.00072	4.416	<0.001
	BV for AFR	-0.00073	0.00027	2.727	0.007
	Phase 2	-0.00086	0.00074	1.166	0.247
	Phase 3	-0.00140	0.00091	1.530	0.130
	BV for AFR x Phase 2	0.00033	0.00029	1.131	0.260
	BV for AFR x Phase 3	0.00098	0.00046	2.129	0.035

Full models used to assess the links between individual age at first reproduction (AFR, (a) and breeding values (b) for age at first reproduction (BV for AFR) on lifetime individual contribution to population growth (calculated as the sum of yearly individual contribution during a women lifetime). Estimates are from linear mixed effects models including year of birth as random effects. Phase 1 was used as the reference category; see Supplementary Fig. 1 and Supplementary Note 1 for additional explanations of the three demographic phases. To assess the significance of the interaction between AFR and phase, we used F ratio tests, and neither interactions were retained (AFR model, p=0.621, BV for AFR model, p=0.108).

Supplementary Table 2. Effects of age at first reproduction and demographic phases on lifetime individual contributions.

	Variables	Coefficients	SE	T-values	P-values
(a)	Intercept	0.00233	0.00049	4.801	<0.001
	BV for AFR	-0.00047	0.00013	3.772	<0.001
	Phase 2	-0.00058	0.00051	1.134	0.260
	Phase 3	-0.00100	0.00069	1.461	0.147
	AFR x Phase 2	0.00009	0.00013	0.678	0.498
	AFR x Phase 3	0.00035	0.00020	1.719	0.087
(b)	Intercept	0.00217	0.00045	4.841	<0.001
	BV for AFR	-0.00037	0.00004	8.594	<0.001
	Phase 2	-0.00042	0.00047	0.872	0.385
	Phase 3	-0.00136	0.00059	2.316	0.023
	Deviance explained				17.3%

Full (a) and reduced models (b) used to assess the links between breeding values for age at first reproduction (BV for AFR) on lifetime individual contribution to population growth using the subfecundity dataset (see Methods on data filtering). Estimates are from linear mixed effects models including year of birth as random effects. Phase 1 was used as the reference category; see Supplementary Fig. 1 and Supplementary Note 1 for additional explanations of the three demographic phases. F ratio test suggests that the interaction between BV for AFR and phase was not significant (p=0.215).

Supplementary Table 3. Association between lifetime individual contributions through survival and reproduction to population growth and breeding values for age at first reproduction.

	Variables	Coefficients	SE	T-values	P-values
(a) Survival	Intercept	0.00048	0.00005	10.181	<0.001
	BV for AFR	0.00010	0.00003	3.924	<0.001
(b) Reproduction	Intercept	0.00175	0.00016	11.03	<0.001
	BV for AFR	-0.00049	0.00008	5.95	<0.001

Effects of breeding values for age at first reproduction (BV of AFR) on individual contribution to population growth estimated considering only the survival (a) or the reproductive (b) component of fitness for cohorts of women born in 1772-1880 at île aux Coudres, Canada. Estimates are from linear mixed effects models including year of birth as a random effect.

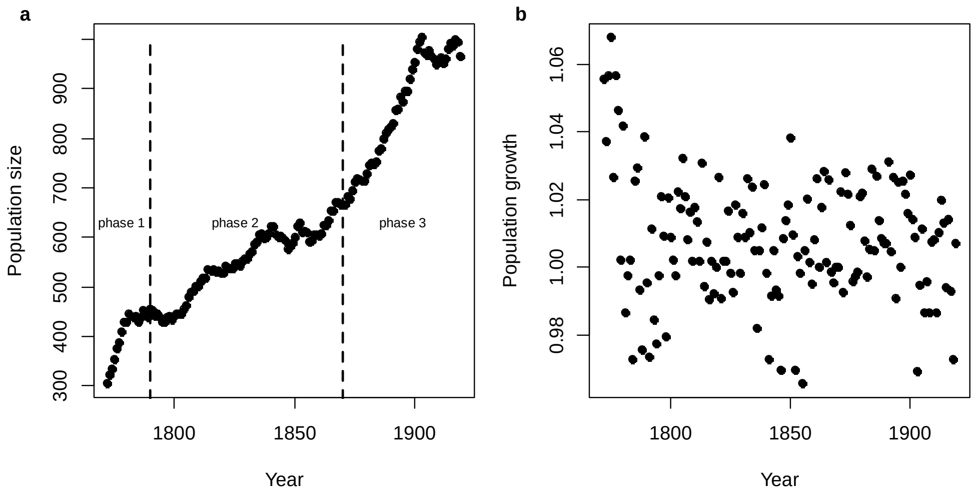

Supplementary Figure 1. Dynamics of the île aux Coudres population. Total number of individual (a) and population growth (calculated at N_{t+1}/N_t) (b) on the île aux Coudres population We restricted our analyses to women born from 1772 to 1880 (see Methods). The dashed lines correspond to the three demographic phases; see Supplementary Note 1 for additional details on the population history.

Supplementary Notes

Supplementary Note 1: *Description of île aux Coudres population and demographic periods*

Île aux Coudres is a small island (34 km^2) in the St. Lawrence River, approximately 80 km northeast of Quebec City. Until at least the 1950s, the île aux Coudres population was entirely Catholic and livelihoods were mostly based on subsistence farming and fishing[1,2,3,4,5,61]. As birth control was not used until the 1950s, the island experienced a natural fertility regime[2,3]. The onset of reproduction began with marriage and its termination by menopause or death of either spouse[2,7]. Birth rates were high throughout the study period (annually 60 births/1000 inhabitants early on and oscillating between 31 and 46 births/1000 inhabitants between 1781 and 1950)[5].

Until 1760, Québec (still New France at the time) was under the *Seigneurial* Regime where the management of the land was divided among *seigneurs* (lords) by the French Crown. Locally, the lord lent parcels of land to peasants who lived on those parcels with their family. Since its settlement, the island's population underwent three main demographic periods according to Martin[5]. First, it increased to around 600 by 1790, then a saturation of available land led to a period of relative stability with a slower growth until around 1870, followed by a period of 'expansion' and steady growth to about 1,600 inhabitants by 1950 (Supplementary Fig. 1a). The first period of rapid growth was mostly due to immigration. By 1765, 41 families were established on the island[5]. Afterwards, the limited resources, mainly available land, slowed immigration. The distribution of non-

reserved land was nearly over and when lords conceded their own parcel on the island, they did so for the benefit of inhabitants' sons[5]. From 1765 to 1790, rapid population growth was mostly due to reproduction. Families established before 1765 continued the settlement, when fathers conceded all or part of their own land parcels to their sons[5]. By 1790, all the land was conceded and population growth declined. During the relative stability, from 1790 to 1870, population growth was still high; however, emigration became frequent[5]. People who emigrated generally did so between the ages of 20 and 25, most often when they married[5]. From 1870 to 1950, the population more than doubled due to economic diversification. A detailed description of the history of this population can be found in [5] and [7].

Supplementary Note 2: ***Example of the approach used to simulate population growth rate in the absence of evolution***

Step 1: Calculation of individual contributions to population growth

The following is a small fictive example of the method used to simulate population growth rate in the absence of evolution. We first generate a small population which is followed for 3 years (Supplementary Figure 2), going from 5 individual to three individuals over 3 years. The size of the population can be obtained by counting the number of unique individuals present each year.

```
library(lme4,dplyr,ggplot2)

dt <- dt %>% group_by(yr) %>% mutate(Nt = length(unique(ID)))

dt <- dt %>% group_by(yr) %>%
  mutate(sbar = sum(surv) / Nt,
         rbar = sum(recru) / Nt,
         pti = ((surv - sbar) / (Nt - 1)) + ((recru - rbar) / (Nt - 1)) )

dt <- dt %>% group_by(ID) %>%
  mutate(LT.pti = sum(pti)) # calculate lifetime individual contributions
```

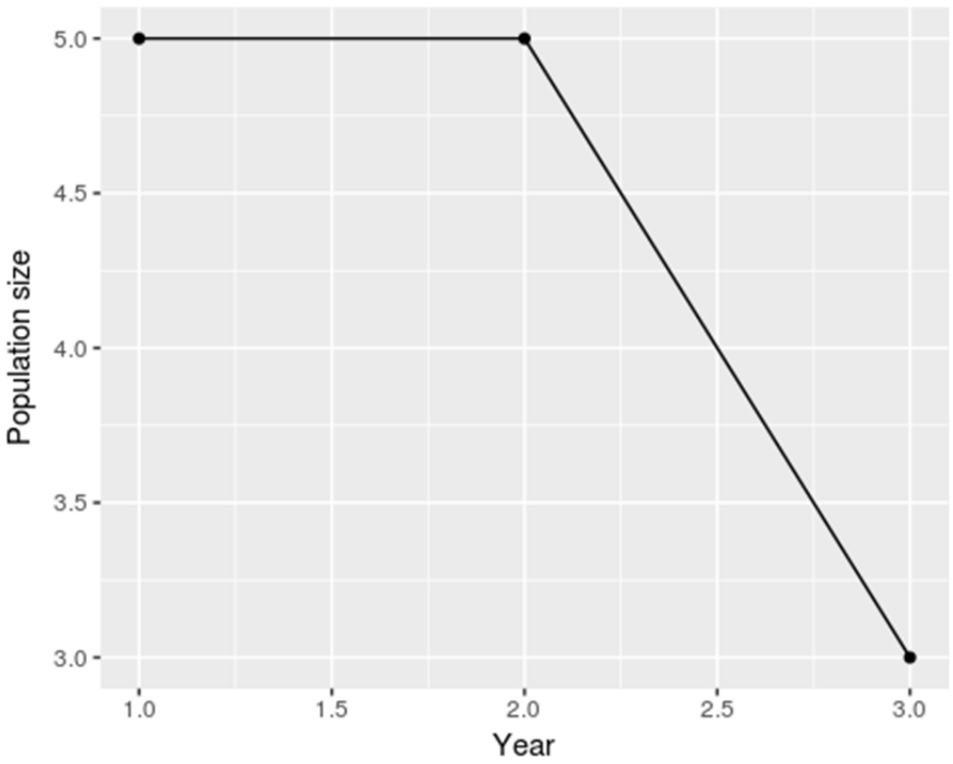

Supplementary Figure 2. Illustration of population size as a function of time for a fictive population monitored for 3 years.

Based on these data, we can determine if each individual survived until next year and whether they produced an offspring which survived until next year (column $s_{t(i)}$and $r_{t(i)}$ in the Supplementary Table 4). A first step in our analyses, is to calculate the mean survival and mean recruitment of the population each year (column $\overline{s}_t$and $\overline{r}$ in the Supplementary Table 4). The individual contribution to population growth rate can then be calculated (column$p_{t(i)}$ in the Supplementary Table 4) using the following R code.

Supplementary Table 4. Life-history table of a small fictive population. Variables are defined as follows: $s_{t(i)}$ survival of an individual i into the next year; $r_{t(i)}$ production of a recruit the by an individual i; N_t population size; $\overline{s}_t$ yearly mean survival; $\overline{r}_t$ yearly mean recruitment; $p_{t(i)}$ individual contributions to population growth; LT.pti lifetime individual contributions (calculated as the sum of $p_{t(i)}$).

year	ID	Cohort	$s_{t(i)}$	$r_{t(i)}$	N_t	$\overline{s}_t$	$\overline{r}_t$	$p_{t(i)}$	LT.pti
1	1	1	0	1	5	0.4	0.600	0.000	0.000
1	2	1	1	0	5	0.4	0.600	0.000	-0.150
1	3	1	0	1	5	0.4	0.600	0.000	0.000
1	4	1	1	0	5	0.4	0.600	0.000	-0.233
1	5	1	0	1	5	0.4	0.600	0.000	0.000
2	2	1	0	0	5	0.2	0.400	-0.150	-0.150
2	4	1	1	0	5	0.2	0.400	0.100	-0.233
2	6	2	0	1	5	0.2	0.400	0.100	0.100
2	7	2	0	0	5	0.2	0.400	-0.150	-0.150
2	8	2	0	1	5	0.2	0.400	0.100	0.100
3	4	1	0	0	3	0.0	0.667	-0.333	-0.233
3	9	3	0	1	3	0.0	0.667	0.167	0.167
3	10	3	0	1	3	0.0	0.667	0.167	0.167

Step 2: Relationship between lifetime individual contributions and breeding value

The lifetime individual contribution is calculated as the sum of all the $p_{t(i)}$ of each individual. Only individuals with known dates of birth and death are used in the calculation of lifetime individual contribution. Then, each individual value is associated with its breeding value (EBV) as calculated from the animal model.

```
tmp2 <- dt %>%
  filter(ID %in% dt$ID[dt$surv==0]) %>% # only keep the dead
  group_by(ID, cohort) %>%
  summarise(LT.pti = sum(pti)) %>%  # sum pti for each individual
  left_join(dt.EBV, by = c("ID", "cohort")) # join LT.pti with EBV data frame
```

The relationship between lifetime individual contribution and EBV is obtained using a Gaussian mixed effect model where lifetime individual contribution is the response variable, EBV is the explanatory variable and year of birth is used as random effect.

```
model <- lmer(LT.pti ~ EBV + (1|cohort), data = tmp2)
```

Supplementary Table 5. Table of the parameter estimates for the fixed effects of a mixed model of lifetime individual contribution to population growth as a function of estimated breeding values.

year	ID	Cohort	$s_{t(i)}$	$r_{t(i)}$	N_t	$\bar{s}_t$	$\bar{r}_t$	$p_{t(i)}$	LT.pti
1	1	1	0	1	5	0.4	0.60	0.00	0.00
1	2	1	1	0	5	0.4	0.60	0.00	-0.15
1	3	1	0	1	5	0.4	0.60	0.00	0.00
1	4	1	1	0	5	0.4	0.60	0.00	-0.23
1	5	1	0	1	5	0.4	0.60	0.00	0.00
2	2	1	0	0	5	0.2	0.40	-0.15	-0.15
2	4	1	1	0	5	0.2	0.40	0.10	-0.23
2	6	2	0	1	5	0.2	0.40	0.10	0.10
2	7	2	0	0	5	0.2	0.40	-0.15	-0.15
2	8	2	0	1	5	0.2	0.40	0.10	0.10
3	4	1	0	0	3	0.0	0.67	-0.33	-0.23
3	9	3	0	1	3	0.0	0.67	0.17	0.17
3	10	3	0	1	3	0.0	0.67	0.17	0.17

Step 3: Back transform to population growth

To quantify the effect of evolution on population dynamics, we simulated a population with no evolution. We used the previously parametrized model to predict lifetime individual contribution for all individuals, given the mean EBV of the first cohort.

```
newd <- na.omit(tmp2[, c("ID", "cohort", "LT.pti", "EBV")]) # obtain prediction data.frame
newd$EBV <- mean(tmp2$EBV[tmp2$cohort==1], na.rm = T) # use mean EBV of first cohort
newd$PredNoEvo <- predict(model, newdata = newd, type = "response",
```

```
                          re.form = ~ (1|cohort), allow.new.levels = T) #predict response
newd$noEvoPLUSres <- newd$PredNoEvo + resid(model) # add residual
```

As our analyses predict lifetime value of individual contributions, we back transformed it into an annual value by dividing it by the number of years each woman was assumed to be alive and present on the island, distributing it evenly through each woman's lifetime.

```
t4 <- as.data.frame(table(dt$ID))
tmp3 <- dt %>% left_join(newd[, c("ID", "noEvoPLUSres")], by = "ID") %>%
  left_join(t4, by = c("ID"= "Var1"))
tmp3 <- tmp3 %>% mutate(pti_noEvo = noEvoPLUSres / Freq)
```

Population growth can be measured as (Nt+1/Nt), but is also equal to the sum of the mean survival and the mean recruitment. The sum of annual pti is equal to zero by definition. The sum of the predicted $p_{t(i)}$pti without evolution is therefore the deviation from observed population growth produced by removing evolution. To obtain the predicted population growth rate without evolution, the sum of $p_{t(i)}$ in each year can therefore be added to the known population growth rate in that given year.

```
tmp4 <- tmp3 %>% group_by(yr) %>%
  summarise(Nt = mean(Nt),
          wbar = mean(sbar) + mean(rbar),
          wbar_noEvo = mean(wbar) + sum(pti_noEvo) )
```

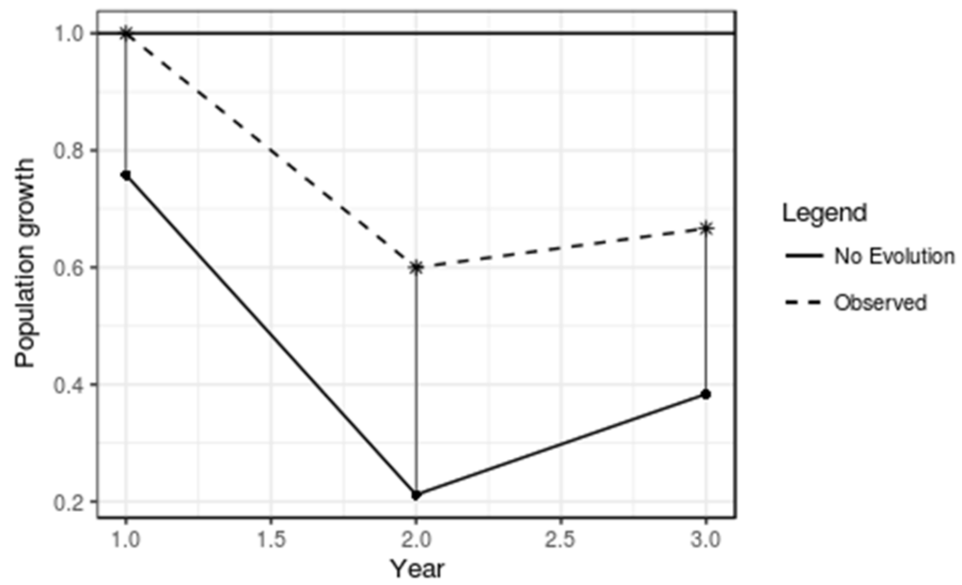

Supplementary Figure 3. Illustration of population growth rate as a function of time for a fictive population followed for 3 years. Solid circles and the full line represent the growth rates (Nt+1/Nt) calculated from the Register, while asterisks and the dashed line represent an approximation of the predicted growth rates, in the absence of evolution.

Supplementary References

[1] Philippe, P. *Inbreeding Structure at Ile-aux-Coudres* [in French]. PhD thesis (Université de Montréal, 1969).

[2] Boisvert M., Mayer F.M. Infant mortality and consanguinity in an endogamous population in Québec [in French]. *Population* **49**, 685-724 (1994).

[3]Philippe, P. Statistical analysis of marriage - first birth and interbirth invervals at Ile-aux-Coudres [in French]. *Population* **28**, 81-93 (1973).

[4]Philippe, P. Amenorrhea, intrauterine mortality and parental consanguinity in an isolated French Canadian population. *Hum. Biol*. **46**, 405-424 (1974).

[5]Martin, Y. Ile-aux-Coudres: Population and economy [in French]. *Cahiers de Géographie* **2**, 167-195 (1957).

[6]Le Querrec, J. Ile-aux-Coudres: Towards an ethno-ecological divorce? [in French] *Anthropologie et Sociétés* **5**, 165-189 (1981).

[7]Milot, E. *et al.* Evidence for evolution in response to natural selection in a contemporary human population. *PNAS* **108**, 17040-17045 (2011).
